# Teaching and Learning in COVID-19 Lockdown in Scotland: Teachers’ Engaged Pedagogy

**DOI:** 10.3389/fpsyg.2021.733633

**Published:** 2021-12-08

**Authors:** Tracey Colville, Sarah Hulme, Claire Kerr, Daniela Mercieca, Duncan P. Mercieca

**Affiliations:** School of Education and Social Work, University of Dundee, Dundee, United Kingdom

**Keywords:** COVID-19 lockdown, learning and teaching, engaged pedagogy, bell hooks, Scotland

## Abstract

This paper reports on a study of teachers’ perceptions of teaching and learning in Scotland during the COVID-19 pandemic through the lens of engaged pedagogy and the ideas of bell hooks. It aimed to explore the different ways that teachers experienced teaching and learning during this time and the impact this may have had on teacher identity. Sixty teachers and head teachers were interviewed using MS Teams in the period April-June, 2020. For this paper, 18 transcripts were analyzed by members of the research team. Four key themes emerged from the interview data: Working from home, parental engagement, teacher identity, and changes in pedagogy. Each of these themes were discussed in terms of concepts such as engaged pedagogy, agency, self-actualization, recognition and boundary transgression situated in the work of bell hooks. The idea of boundaries wove itself throughout our data as teachers expressed how the transgression of boundaries was occurring in multiple, and often contradictory, ways in pedagogical, professional, institutional and personal spaces and systems. We see in our data evidence of a shift in practice not just in the way teachers are ‘doing’ education but also, perhaps, in the way that teachers are ‘being’ as educators as they adapt to different ways of knowing. This study provides a unique exploration of a time and space in Scotland during 2020. However, the themes and understandings that emerged are of relevance to educators internationally. Schools across the world were impacted by various lockdowns imposed by the Covid-19 pandemic and teachers faced a common set of challenges that were resolved via re-negotiation and recognition of individual and collective agency to create new pedagogies.

## Introduction

Schooling is a fundamental part of society’s fabric. The COVID-19 pandemic and its lockdowns have offered a dilemmatic context where the processes of teaching and learning and being a teacher have been re-visited and re-negotiated. This paper draws from a study which was carried out during the first COVID-19 lockdown in Scotland that looked at the lived experiences of primary school teachers and Head Teachers of teaching and learning during this time. It offers a unique insight into the lived experiences of teachers – how did they manage/adapt and what was important to them. It was stated by the Scottish Government (2020a,b), that teaching, learning and support during school lockdown would continue but in a different way, with teachers mostly connecting with pupils through distance and online learning. Although the research was based in Scotland, the themes are of international relevance to teachers working across the world, who have all been impacted by the global pandemic. This paper explores how teachers experienced these different ways of teaching and learning, and asks, what was the teacher’s experience during this lockdown?

The next section concerns both the literature and national context in which this research project was carried out, followed by an outline of the methodology which framed this research project, the analysis of the data, and the write up of this particular paper. We consider the relevance of the ideas of bell hooks as the theoretical framework within which the interviews were analyzed, and from which four themes emerged: Change in Pedagogies, Agile and Flexi-working, Teachers’ Identities, and Parental Engagement.

### Teaching and Learning During the COVID-19 Pandemic Lockdown

As researchers internationally have sought to capture the experiences of both teachers and pupils in this unique set of circumstances, literature is starting to emerge which seeks to explore different impacts that the lockdown has had for different groups across a range of areas. As some point out, the lockdown provided the circumstances for ‘a shocking, and at many times, painful natural experiment’ (Jones and Kessler, 2020, para 3) that many researchers have endeavored to capture.

A range of areas relevant to teaching and learning have been reported so far. Authors have focused on the challenges of incorporating a digital pedagogy into the learning environment (Wong, 2020; Greenhow et al., 2021), teacher identity (Jones and Kessler, 2020) as well as changes in practice and teacher reactions that the lockdown necessitated (Collie, 2021). Much of the current literature seeks to understand the unique space that teachers found themselves in during online teaching and the tensions that teachers have found themselves having to navigate between systems, identities, practice, and pedagogy. For the purposes of this paper, we have focused on capturing the experiences of teachers in Scotland, and have reported on the literature in more depth in the relevant sections of our theory led thematic analysis as per this model.

### Scottish Context

During the first pandemic lockdown, the United Nations Policy Brief [6] in April 2020 reported that 188 countries had imposed nationwide school closures, affecting more than 1.5 billion children and young people (p. 2). This led to uncertainty and tentative planning with unknown timescales. In Scotland, all schools were closed on 20 March by the Scottish Government on advice of the Scientific Advisory Group for Emergencies (SAGE). Exceptions were for vulnerable children or children of key workers who attended “hubs” run by volunteer teachers. Schools re-opened on 11 August, 2020 with phased returns to school for different age groups. By late December, 2020 Scotland entered the second lockdown with another phased return on 25 February, 2021. The time period for interviews in this study was March – June, 2020; during the first lockdown. Participants were teaching on-line or in education hubs at the time of interviews.

Following the abrupt disruption of schooling caused by closures, teachers had the challenge to find alternative and innovative ways to reach and teach all children and young people. Support services such as Educational Psychology had to find new ways to support school, families, and children. This led to distance and online learning with as much continuity as possible, including on-line Getting It Right For Every Child (GIRFEC) meetings. In Scotland the GIRFEC framework (Scottish Government, 2006), which embodies the UNCRC (United Nations Convention for the Rights of the Child), promotes good practice in supporting the wellbeing of children and aims for young people to receive “the right help, at the right time, from the right people” (Scottish Government, 2008). For most learners this support moved online unless they were vulnerable or had parents who were keyworkers. In such instances they were supported via education ‘hubs.’ A range of guidance, national and local was published to support practitioners move their practice online with confidence (British Psychological Society, 2020; Education Scotland, 2020; Scottish Government, 2020a,b). This guidance aimed to scaffold equity of support for children and their families including those with Additional Support Needs (ASN) in relation to digital teaching and learning. In addition, many parents found themselves in the position of working from home and home schooling. The Scottish Government stated that parents would not be expected to engage with their children’s learning formally nor to act as teachers.

The nature of the educational infrastructure in Scotland meant that this guidance could be interpreted and enacted differently at local authority and school level. Responding to this shift, schools and staff developed new models of service delivery, so that there was variation both across and within authorities in terms of format and speed of change. Schools that had previously embraced the national digital technology strategy were more prepared for this rapid shift. The most commonly used digital platforms within Scottish education during the initial lockdown were the national intranet GLOW, Microsoft Teams, Google Classroom, and the Seesaw App.

The teachers interviewed in this research offered narratives of their lived experience of teaching online during this time and their individual experiences reflect the flexibility of how guidance and change were implemented within their local authority. Some practitioners were involved in providing both face to face teaching in ‘hubs’ for vulnerable or keyworker children and online teaching for their own classes. At this point in the pandemic the teaching experience was focused upon reinforcement and consolidation of the key areas of literacy, numeracy and health and wellbeing. Alongside this many of our interviewees were additionally home schooling their own children. The researchers of this study were academic researchers and educational psychologists some of whom were supporting schools and staff at this time and thus had insight into the narratives being heard.

## Methodology

As past teachers and currently trainers of teachers and educational psychologists, indeed, as parents of children who attend schools, we were acutely aware of the flurry of activity going on in schools in March 2020 as it became increasingly clear that school closure was imminent. The experience of confusion and uncertainty motivated a desire to capture others’ experience of this, especially others involved in education. There was recognition that this was unprecedented and that we wanted to pause and document the experiences of a group of professionals (teachers) whose working lives, and indeed, whole pedagogical approaches had been disrupted by the pandemic. We were aware that time was a factor and that this particular set of circumstances was boundaried in that it would evolve quickly. We were therefore under a pressure of time to capture as many experiences via interview that we could.

We, the authors, are part of a larger group of researchers from the School of Education and Social Work at the University of Dundee, all of whom were keen to capture the impact of this unique time on the lives of children, their families and educators. A decision was taken to carry out qualitative research with teachers and Head Teachers working in Scottish primary schools, focusing upon narratives and detailed insights into the lived experiences of teachers who had volunteered for interview. We could not ethically gather more data than we could realistically manage to analyze, although the availability of it was tantalizing. Reluctantly, we made the decision not to pursue the stories of educators in secondary schools or those of families and children themselves for this particular study but recognize the need for further research with these groups. The research leads of this project came up with questions which they thought could prompt conversations covering the whole experience of the sudden lockdown and the slow realization of the implications of the lockdown. As Hayes writes about her research, “there were interviews which took the form of conversations, but in which I made sure that the conversation covered half a dozen specific points” (p. 103). These questions were reviewed by critical colleagues interested in supporting the project. Also, the research teams through their contact with teachers asked for teachers working in primary contexts who were willing to give feedback on interview questions from their perspective. Two primary teachers offered, and feedback was given as to the relevance of the questions, the language used, and the sensitivity of the issues raised. These were not considered as pilot interviews, and therefore not included as data. The questions are listed as an Appendix at the end of this paper.

A decision was taken to carry out qualitative research with teachers and Head Teachers working in Scottish primary schools, focusing upon narratives and detailed insights into the lived experiences of teachers who had volunteered for interview. We opted to interview primary school teachers (from Primary 1 to Primary 7) and Head Teachers of Primary schools for two main reasons. Second, most of the research teams were primary school teachers themselves, before training into other professions such as educational psychology or teacher educators. Reluctantly, we made the decision not to pursue the stories of educators in secondary schools or those of families and children themselves for this particular study, but we recognize the need for further research with these groups.

Following ethical clearance from the University of Dundee, teachers and Head Teachers in Primary schools in Scotland were invited through two social platforms: Facebook and Twitter to participate in an in-depth oral interview to capture their lived experiences of the pandemic lockdown in real time. Sixty teachers contacted the lead researcher and volunteered to participate in the research. These online interviews were carried out through MS Teams, starting from the second week of lockdown (March 2020) until the end of the scholastic year in June 2020. The interviews lasted between 30 and 90 min. We are aware that this implies teachers and Headteachers who had access to technology and access to these two platforms could get information about the research. Given that the research started from the second week of the first lockdown, social media platforms were the only way we could communicate as schools and Local Authorities were closed.

The research aimed at gathering teachers’ understanding of their experiences and the impact of these on how they viewed their work and role as teachers. What were teachers’ perceptions of their current experiences of work and how did this reflect on what they valued in their teaching and learning as they supported children and families during the lockdown? The participants who volunteered to be interviewed reported that they relished the opportunity to pause and reflect on their experience and also to appreciate the numerous ways in which they adjusted to the changes in their lives and their practice in a short span of time and under intense pressure. Nine questions guided the interviews, with the interviewers reporting that most participants needed little prompting as they were very articulate and fluent.

It is important to point out that due to the urgency of the research, it was not possible to delay the start of data collection until a proposal could be submitted to apply for research funding, which would have covered costs of making the recorded data ready for analysis.

The research group was not seeking a representative sample of educators which was distributed according to certain criteria. The aim was not to gather a sociological understanding of teachers’ experiences based on the geographical and socio-economic contexts of the Scottish schools in which they worked, nor to collect a representative sample of educators and elicit collective themes from the corpus of data which could be claimed as generalizable. Rather, we aimed to capture the educators’ personal thoughts about these experiences. We recognize that as we did not stipulate conditions of participation (e.g., time in teaching, or age group of class) then we would not be able to comment on how such conditions might interact with the experiences of the participating teachers. However, this was not our aim. Instead, we hoped to capture the immediate and lived experience of those primary teachers who felt able to participate at a point in time during the first lockdown. As Beauchamp et al. (2021) pointed out about their own research during the pandemic, the circumstances surrounding our research project lent an urgency to our gathering of the educators’ recounting of their experiences. We took the steps outlined above to ensure that our questions and data gathering process was robust and sensitive to participants. Given the time sensitive nature of the research project, we aimed to gather the verbal responses of any teacher or Head Teacher that volunteered to be interviewed, including all those who met the inclusion criteria of teaching in a primary school in Scotland. The number of volunteers happened to be sixty and we decided to form groups of researchers, taking a small number of interviews each group, and to analyze these interviews according to the specific slant that the group decided on. This decision was arrived at following several conversations about the interviews among the group members.

We were aware that this approach would not adhere to a traditional scientific conceptualization of generalizability (Williams, 1976; Law, 2006) however, we decided to align ourselves with the work of Hollway (1989) who suggests that “generalizability has to be established according to theoretical rather than statistical principles” (p. 16). In the Rogerian sense of what is most personal is most general, we believe that the issues emerging from our process of analysis (explained below) are generalizable conceptually and theoretically. When disseminating our research findings in online seminars, we have had feedback that viewers have resonated with the participants’ expressed thoughts and feelings (Rogers, 2001, p. 26).

Out of the 16 interviews, eight groups of researchers have so far been looking at different interviews with different theoretical frameworks. This paper reports on one of these groups. Other papers have so far been published on Head Teachers’ understanding of care and how this informed their actions during the closure of schools (Ferguson et al., 2021), the reciprocal caring relationships between teachers, children and parents in those first weeks of school closure (McLennan et al., 2020), and teachers’ responses to the increased need to engage with digital technology during the first lockdown (Brown et al., 2021). We see the results emerging from each paper as complementing each other, while each one has its unique stance on the data. For this paper, we drew upon eighteen interviews, most of which were conducted by the authors themselves as the familiarity with the text lent itself to the analysis. The focus of this paper is on teaching and learning during COVID-19 school lockdown from the perspective of teachers – a focus yet unexplored from the data. We also used a theoretical framework derived from bell hooks’ work on engaged pedagogy which stands on its own in relation to the different theoretical frameworks that we have used in other papers that have derived from this research. The methodology of all the papers is similar and therefore we acknowledge that there are moments when this is very similar to the other papers.

### Theory-Led Thematic Analysis

In qualitative research, themes can be derived inductively (empirically induced from the data) or deductively from prior theorizing (*a priori* themes) (Cohen et al., 2017; Silverman, 2017). *A priori* themes may be based upon knowledge of the evidence-base or literature around the topic under investigation, from authors’ theoretical orientations and previous empirical research, and from everyday constructs (Denzin and Lincoln, 2011; Corbin and Strauss, 2014). Many researchers will use both techniques to align with theoretical positioning and to derive themes empirically from the data (Miles et al., 2019). While this paper used a theory-led thematic analysis, the authors are aware of the limitations of both approaches. In being theory-led, formation of new ideas and connections may be missed and there is a risk of finding only what you are looking for in the data, conversely, lack of theory in thematic development limits connections between empirical data and the research questions (Creswell and Creswell, 2018). The approach used in this paper is influenced by the work of Hayes (1997) who uses theory-led analysis. Two of the authors of this paper have also used this approach in several publications.

The process of analysis can be visualized in the form of two spirals which seemed to weave closer and closer together as they progressed, one spiral being the work on bell hooks and the other is the interviews. Following an initial discussion in which impressions of carrying out interviews were shared, it was decided that, in the first instance, each researcher would analyze one interview transcript individually and separately. Following this activity, the researchers discussed recurring points and emerging themes, formulating a tentative guide around which we could analyze other interviews. Thirteen over-arching themes were identified in total with the initial themes being built upon with each subsequent interview analysis. Due to the volume of data it was necessary to be selective in our focus for this paper. Four themes were selected that yielded the largest volume of data and were thought to best represent the overall experiences of the teachers/headteachers in relation to learning and teaching. From these, sub-themes and illustrative quotes were derived which gave voice to the over-arching themes and made direct links between the raw interview data and the researcher derived theme. An illustration of this within the ‘Parental Engagement’ theme is the link of the sub-theme of ‘collaborative approach with parents’ and the quote from a teacher ‘We’re kind of shepherding each other through, cause we’re a whole flock together and that’s how we need to get through this.’ We chose 18 interviews, most of which were conducted by the authors themselves – the richness of each interview contributed amply to each theme and satisfied our aim of conceptual and theoretical generalizability mentioned above. The authors of this paper initially took one interview analyzed it collectively. The interviews were then divided amongst the authors for analysis. Several meetings took place during this process to check out that the thematic analysis was reliably done by all of us and there was consistency in the process and analysis. Also, these meetings helped us support each other in reading about bell hooks and interpret the data in the light of hooks’ ideas. In this way, our discussions could be seen as taking part within our own evolving understanding of the unique and unprecedented context that the COVID-19 pandemic brought about as we sought to understand others’ experiences also. This was a dynamic process and akin to Wenger’s (1998) conceptualization of a Community of Practice. Our desire to make sense of what was happening was linked to our practice, as educators and with educators, in exploring the meanings that we jointly created as a community of researchers.

Our discussions also led to a consideration of the work of bell hooks, an American author writing under the lowercase pseudonym of her great-grandmother’s name. Her writing about transgression against and beyond boundaries struck us, as we felt it resonated the freedom which ensued following with the removal of physical boundaries due to the closure of schools. Since a key theme that emerged from the data was the freedoms and changes in relationships between teachers, young people and families, we decided to pursue bell hooks’ writing on ‘opening boundaries’ and ‘engaged pedagogy’ to inform our analysis of how teachers described changes in interactions and power dynamics caused by the COVID-19 pandemic. The barriers imposed by structures of the education system and cultural expectations for achievement were suspended and there was tentative, and later, more bold exploration of what was possible. Interestingly, the pandemic brought with it new boundaries and restrictions in people coming together, and there seemed to be a sense of ‘we are all in this together’ as teachers shifted from their customary role of class teacher causing personal boundaries to blur as they managed the stresses of the restrictions on their lives and the present unknown. As bel hooks stated:

I cross boundaries to take another look, to contest, to interrogate, and in some cases to recover and redeem (hooks, 1994b, p. 5).

The two spirals were further woven as the authors read further about the works of bell hooks, while at the same time becoming more and more immersed in the data. As Hollway and Jefferson (2000) stress, “after a whole day working on the transcripts … (a process we often referred to as ‘immersion’) we would be inhabited by that person in the sense that our imagination was full of him or her” (p. 69). The authors met online several times to discuss their thoughts about the literature and the interviews, with themes becoming more tangible, following Hollway’s idea that the significance of the interviews is not only “a property of the extract, but of the work it is put to do” (Hollway, 1989, p. 36). Hayes also writes about this process in her edited book, where she mentions “theory-driven themes, rather than the analysis being based on themes which arose spontaneously from the data” (p. 99). This was thus a theory-led thematic analysis (Hayes, 1997) based on the works of bell hooks, where both theory and interviews ‘speak’ to each other while at the same time challenging each other. This influenced the structure of this paper, with no distinction between findings and discussion, but rather themes with merging data and theory, thus ‘making complex’ the lived experiences of the teachers. There is always the tension of how the data and theory speak to each other – what influences what in developing the themes. We read the transcribed interviews and reading hooks simultaneously. It was through a continual conversation between us authors that we felt that we needed to focus on four main themes as will be described below.

A last point about interviewing educators who volunteered: we are aware of the possibility of these respondents presenting as models of hard work and enthusiastic professionalism. We have mentioned elsewhere that these need to be seen as experiences of people willing to share their story and that there are others whose stories may not tally. Yet, there have been many who have ‘recognized’ our interpretations and analyses, that is, the sense that we made out of them can be shared through the subjectivity of others (Hollway and Jefferson, 2000, p. 80). Our work, as well as being theoretically led, is solidly empirical in the sense that supporting and challenging evidence is available (Hollway and Jefferson, 2000, p. 80).

### Engaged Pedagogy: Introducing Bell Hooks’ Ideas

For those who have not engaged with the writings of bell hooks, it is important to start with a reflection of the name *per se*. Born Gloria Jean Watkins in 1952, bell hooks grew up in segregated Kentucky (United States) in a nuclear family of five sisters, a brother, a mother and a father, with nearby extended family. *Ain’t I a Woman: Black Women and Feminism* (hooks, 1981) was written when she was a 19-year-old undergraduate. Her great-grandmother was ‘a sharp-tongued woman, a woman who spoke her mind, a woman who was not afraid to talk back’ (hooks, 1989, p. 9). Hooks put the name in lowercase letters not only ‘to distinguish [herself from] her great-grandmother,’ but because she wants readers to focus on the ‘substance of books, not who I am’ (Williams, 2013). Her renaming was itself ‘a gesture of defiance that heals, that makes new life and new growth possible’ (hooks, 1989, p. 9). As she notes, “choosing this name as a pseudonym was a rebellious gesture’ (hooks, 1989, p. 163). The act of the name thus signifies an act in reconstituting and reinventing her identity (see Guadalupe Davidson and Yancy, 2009). The issue of challenging and reconstituting one’s identity is a fundamental aspect of hooks work and will be discussed further in the theme on Teachers’ Identities.

The focus of hooks work seems to be on opening ‘boundaries,’ ‘transgressing boundaries’ (hooks, 1994a, p. 13), ‘crossing boundaries’ (hooks, 1994b, p. 5) and ‘movements of ideas, exchanges by everyone’ (hooks, 2009, p. 21). Hooks words capture this movement between boundaries:

I celebrate teaching that enables transgressions – a movement against and beyond boundaries. It is a movement which makes education the practice of freedom (hooks, 1994a, p. 17).

The crossing of boundaries implies freedom, or at least envisages ways that such freedom of movement that can be experienced by everyone. Spaces, for hooks, are political. They need to be revolutionary, as contrasting to oppressed (see Freire, 1972). Yet, spaces are ‘progressive cultural revolution[ary]’ (hooks, 1994b, p. 8) when everyone involved learns to do everything differently, to challenge the politics of domination. The emphasis on ‘everyone’ is a fundamental cornerstone in hooks work. In her work on education (hooks, 1994a,^2003^), it is the student and the teacher that need to transgress/cross boundaries.

Engaged pedagogy begins with the assumption that we learn best when there is an interactive relationship between students and teachers. It requires the active contributions of both being ‘active participant[s], not passive consumer[s]’ (hooks, 1994a, p. 14). Teachers need to find out, to discover what students know and what they want to know (hooks, 2009, p. 19). For hooks this interactive relationship is an intimate one – hooks repeatedly writes that engaged pedagogy ‘respects and cares for the souls of students’ (hooks, 1994a, p. 13). The attention here is on recognition (hooks, 1994a, p. 13) as will be discussed in the theme on Agile and Flexi-Working in the analysis section. The teachers’ and students’ recognition of each other as active participants (hooks, 1994a, p. 14) is a foundational part of the teaching/learning process. There is a ‘will and desire to respond to our unique beings’ (hooks, 1994a, p. 13). This is the Freirean influence on hooks where praxis is understood as ‘action and reflection upon the world in order to change it’ (hooks, 1994a, p. 14).

The issue of ‘knowledge’ is fundamental to the student-teacher relationship as knowledge creates dominance over groups of people. We need knowledge that does not create spaces of domination over others (this will be explored further in the theme around Parental Engagement of the analysis). It is therefore not a question of epistemologically substituting old forms of knowing with new forms of knowledge, ‘but [is about]. learning about and genuinely valuing ways of knowing and understanding.’ Hooks shifts the issue from content to process. The epistemological emphasis should not be about (common) identities and backgrounds, but a shared desire to know:

What we all ideally share [then] is *the desire to learn* to receive actively knowledge that enhances our intellectual development and our capacity to live more fully in the world (our emphasis, hooks, 1994a, p. 40).

It is the intellectual inquiry that counts, and not the communality (or not) of those participating in it. Hooks recommends that everyone (teachers and students as forming a learning community) recognize and learn the ‘cultural codes’ of others. This implies that we ‘learn to accept different ways of knowing, new epistemologies, in the multicultural settings’ (hooks, 1994a, p. 41). Knowledge is a field in which everyone ‘labors’ (hooks, 1994a, p. 137) and no one is excluded.

Engaged pedagogy insists that the teacher has the responsibility to work toward self-actualization, to be aware of themselves as practitioners and as human beings, if they wish to teach students in a non-threatening, anti-discriminatory, empowering way. The focus here is on the dynamic and fluid nature of engaged pedagogy with critical reflection as core to teaching and learning. There are clear links here to Friere and Giroux’s critical pedagogy, to Dewey’s democratic education, Maslow’s self-actualization and the work of AS Neill, the Scottish educationalist who advocated for freedom in the process of learning and teaching as a child-centered approach. Self-actualization, according to bell hooks, should be the goal of the teacher as well as the student although she recognizes that this is not easy:

It was difficult to maintain fidelity to the idea of the intellectual as someone who sought to be whole-well-grounded in a context where there was little emphasis on spiritual wellbeing, on the care of the soul. Indeed, the objectification of the teacher within bourgeois educational structures seemed to denigrate notions of wholeness and uphold the idea of the mind/body split, one that promotes and supports compartmentalization’ (hooks, 1994a, p. 16).

Self-actualization is needed to confront internalized racism, class privilege, and political entitlement in oneself and others. This requires secure, mature emotional skills and a powerful ability to communicate. It is the ability to recognize one’s own privilege and power and address this. The body, mind, emotions are all interrelated for hooks. Her argument is that education tends to focuses on the mind and gives little or no space to the body and emotions. Questioning repressions and denials due to oppression(s) (this could be clearer) helps to develop a feeling for wholeness, that strives for actions, intellect and voice (see hooks, 2009, p. 21). This implies that those involved in such processes are at times exposed and vulnerable. For example, in engaged pedagogy, teachers can face their deep-seated fears about loss of control of the classroom to a community of inquiry, where students, in various ways, bring to the classroom their lived experiences and the knowledges emerging from these.

Four of the authors of this paper are educational psychologists who are or where in practice and who are involved in the training future educational psychologists. This is pertinent as pedagogy is central in the work on an educational psychologist. Yet we are aware that over the years general pedagogy has become a more performative task. All teaching and learning is now being measured and calculated, where criteria and proformas make claims on educators’ attention and time while they are also keen to develop relationships within their educational contexts. bell hooks’ engaged pedagogy offers an alternative voice – a challenging one, but certainly one that opens up possibilities. The data set was particular as it was capturing a unique moment where school builds and classroom were closed, and all the performative and measurable criteria usually leading action lost their place. This allowed different approaches to learning and teaching to be thought about and acted on. Hooks’ work was thus seen as appropriate as it provided us with a language that could help us articulate ideas emerging from the data.

## Thematic Analysis and Discussion

Thirteen over-arching themes emerged from the interviews. These were Technology, Agile and Flexi-working, Staff Collaboration, Managing Transitions, Change in Pedagogies, Parental Engagement, Hubs, Local Authority/Union Stance, Teachers’ Identities, Meeting Needs, Future, Uncertainty and Space. Due to the volume of data it was necessary to be selective in our focus for this paper. Four themes were selected that yielded the largest volume of data and were thought to best represent the overall experiences of the teachers interviewed. Each is explored in relation to the data and also the theoretical framework offered by hooks. These four themes are Change in Pedagogies, Agile and Flexi-working, Teachers’ Identities, and Parental Engagement.

### Theme One: Change in Pedagogies: ‘Extra Creative’

Learning is commonly associated with ‘classroom space.’ Outdoor learning (Education Scotland, 2010) is often seen as an alternative learning approach to classroom learning. While this is gaining more visibility within Scottish schools, classroom-based learning is still the dominant way of providing for learning supported by alternative learning approaches. According to Flores (2020) “the COVID-19 pandemic has changed our everyday life in many ways and, in particular, the education sector” (p. 297). With the arrival of the COVID-19 pandemic, the closure of schools challenged the idea of classroom-based learning as well as student–teacher (face-to-face or the physical presence) interaction, both of which were part of our everyday life. These two challenges are at the heart of hook’s philosophy. For hooks, the classroom is ‘the most radical space of possibility in the academy’ (hooks, 1994a, p. 12). The classroom, with all its limitations, is elevated as a location of possibility, for it offers a possibility for anyone (students, teachers and, to a certain degree, families) to ‘learn’ (hooks, 1994a, p. 13). It is a space where interactions between with two main characters, students and teachers, can ‘transgress those boundaries’ (hooks, 1994a, p. 13). We question how the closure of schools challenged ‘learning’ given that the classroom space was not available? How did the student-teacher interaction manifest itself during the COVID-19 school closure?

Several teachers who were interviewed argued that ‘it’s the happiness and the wellbeing of the children that come first, not the actual learning.’ In this quote the teacher seems to be differentiating between learning and happiness/wellbeing. There seems to be a further assumption that happiness and wellbeing are similar or referring to the same thing, that in any case they are based at the other end of the spectrum to learning. It begs the question: does learning make students (and similarly their teachers) unhappy or unwell?

Many other teachers argued that a different learning was happening:

Yes, children are missing out on learning but I think the learning is taking a different shape and I think it is more that the relationships with families that we have to think about and for what the children really capable of.

The complexity and contradiction in this quote may reflect more generally the experiences of teachers during lockdown. Billig’s concept of *dilemmatic thinking*, when something ‘is and is-not’ is useful in this context (Billig et al., 1988). It is difficult to say what the teacher is implying in this quote when the teacher talks about ‘missing out on learning.’ From our experiences as primary school teachers and now involved in teacher-education, we could infer this to mean learning of literacy and numeracy. We think that the relationship-based learning mentioned in the quote above is very interesting as it is a learning that “is taking a different shape.” The word ‘shape’ here is very evocative, and hooks’ work influences our reading of this. Who shapes this learning? Does the teacher or the student–teacher relationship have this influence? And is this space shaped in a way that allows for ‘freedom’ which is ‘deep and intimate’ (hooks, 1994a, p. 13)? In hook’s words: ‘…our work is not merely to share information but to share in the intellectual and spiritual growth of our children’ (hooks, 1994a, p. 13).

During the first school’s lockdown in Scotland teachers were advised not to give new content material to students but to consolidate learning that they had previously done in schools. Since the first lockdown started with the onset of the spring season, many teachers resorted to the outside space as a source of inspiration and resource: ‘Sometimes going outside picking up stones and sticks that actually can be just as valuable and you don’t need to spend all that time laminating.’ This is always haunted by the question whether this is learning and what sort of learning it is:

Made me [teacher] appreciate how much children can get from activities that a lot of parents don’t see as learning. We don’t need to be sitting down at a table to, you know, to do some writing in order to develop your literacy skills.

One of the terms used by some teachers was contextual learning:

Because it’s putting it into context, it’s putting their learning into context. It makes it memorable as well. Sitting doing a page of maths out of a textbook isn’t memorable learning to me but if you’re doing something, for example, through baking or practical tasks, they, they, they’re more memorable for children.

The focus of memorable learning, as different from ‘rote, assembly line approach to learning’ (hooks, 1994a, p. 13) is brought about by distinguishing between acts of sitting (doing a page of maths out of a textbook) and acts of doing (baking). Another teacher spoke about recording activities for her students on her croft – using her croft as a ‘doing’ context. She shared her lifestyle with students and their families through these video recordings in which she was constantly accompanied by her toddler son, putting into question the professional – personal life boundary. This is revisited in Theme 3 when exploring identities. It is not only the learning which is contextualized but also the teaching. We can read the above teacher’s quote in a romantic Rousseauian way, and it also brings to mind hook’s claim that ‘learning is a place where paradise can be created… In that field of possibility we have the opportunity to labor for freedom, to demand of ourselves and our comrades, an openness of mind and heart that allows us to face reality even as we collectively imagine ways to move beyond boundaries, to transgress. This is education as the practice of freedom’ (hooks, 1994a, p. 207). We couple this quote from hooks with one from our teachers interviewed:

I think this time has allowed me to see lots of different creative ways… like, extra creative,

and with an exhortation by Mushtaque et al. (2021) that since

COVID-19 has the ability to fundamentally reshape our world; … remarkable creativity and student responses must be encouraged, giving educators autonomy and flexibility to work collaboratively (p. 21).

This creative way of ‘doing’ learning and teaching was acting as a catalyst with some teachers to question the systems, structures and performative culture they worked in. Several issues were mentioned from

the climate of worry about taking children out with the school (going to the park or going to go down to the river) – that puts people [teachers] off,

to the fastness of the curriculum:

‘I think that will definitely change when we go back [to schools] be more reflective and how slowly we take things sometimes instead of us teachers always wanting to do everything at once and get all children doing everything.’

The boundaries of what defines the systems, structures and performative culture are being challenged. Maybe to use the word ‘transgress’ as hooks does, might be claiming too much, but certainly there is desire on the part of several teachers interviewed to change, and take this time away from schools and classrooms to reflect, not only on their identity (see Theme 3 in this section) but also on their perspectives of teaching and learning. We feel that the following quote captures this sentiment:

I feel as a teacher that going back, I’m not going back as the person I was before… I feel more, I feel more, I have more ownership. I’ve always felt that I’ve got quite a lot of ownership but I feel I’ve got more ownership and increased sense of ownership.

Often teacher-education and teachers’ discourse is silent on the issue of Eros. Yet, in this sub-theme there are many moments when the Platonic and cartesian distinction between thinking and body remerges. We conclude this sub-theme with a reflection from hooks that encapsulates some of the ideas presented. Hooks constantly reminds us to acknowledge the body, of all those involved in learning and teaching. She posits Eros as that which is other than merely sexual, as that which transforms potentialities to actualities (hooks, 1994a, p. 194). Dealing with our passions makes our lives whole (hooks, 1994a, p. 195). It is through confronting Eros that hooks actually ties in our efforts at self-actualization, and the concepts of engaged pedagogy (being engaged in body, spirit and mind) and liberatory practice.

### Theme 2: Agile and Flexi-Working

Agile and flexible working in the digital age has been subject to academic study prior to the pandemic in terms of new ways of working to meet service and market needs (Grant and Russell, 2020). However, the demands of lockdown necessitated a sudden shift to home working for millions of people and these concepts are now being discussed more generally across society. This period of rapid change, unpreparedness, fear and uncertainty has taken its psychological toll on people with demands to adapt to different ways of working and learning whilst experiencing simultaneously a marked reduction in physical/social contact and travel (Tehrani, 2010; British Psychological Society, 2020). Basile and Beauregard (2020) refer to one of the challenges that agile/flexible working present to work-life balance as an ‘always on’ or ‘switched on’ work culture and the need for clear work/home boundaries to mitigate against negative impact on health and wellbeing.

Professional and public faced guidance documents were produced by the British Psychological Society including advice about working from home and taking trauma-related work home. Specific documents were published for educational contexts (British Psychological Society, 2020). United Kingdom and Scottish Governments also published guidance documents to advise and support the move to remote learning and teaching across early years, primary, secondary, tertiary and higher educational settings [Scottish Government, 2020a,b; Department of Education (DoE), 2021]. Many research studies during the pandemic focused upon its impact on remote teaching and learning from the perspectives of parents, teachers and learners (Kidd and Murray, 2020; Kim and Asbury, 2020; Zhou and Wolstencraft, 2020). Participants in our study recognized advantages and disadvantages of remote working and this is reflected in many findings from studies over the last 2 years (Beattie et al., 2021; Dempsey and Burke, 2021).

The idea of being ‘always on’ is reflected in teachers’ experiences as a disadvantage of homeworking during the pandemic and this is also a concern for hooks. In a very different context, hooks’ shares her narratives and those of other teachers who feel the negative impact of being constantly within classrooms engaging with students and also of navigating schooling systems that can often be ‘racially biased’ (hooks, 2003, p. 17). During the COVID-19 pandemic lockdown, the constant engagement which hooks refers to and which Basile and Beauregard as being ‘always on’ was happening at an intensified level, and on many aspects of teachers’ lives. Hooks’ concept of recognition offers a means by which to resolve such challenges. Recognition, for hooks, is fundamental if we want

to address and resolve issues…[and that it is] needed to generate anew and inspire a spirit of ongoing resistance (hooks, 2003, p. xiv).

In our analysis of the interviews, we noted that some teachers were trying to recognize signs of being hopeful in their teaching and students’ learning during the pandemic. In this theme we identify different ‘recognitions’ that teachers working in Scotland were experiencing.

#### Recognizing Some Benefits and Challenges of Home Working

Several of our interviewee respondents referred specifically to the benefits of working from home during the pandemic. These include undertaking continuing professional development activities, more time to prepare work tasks, more timely assessment feedback, developing skills, less pressure from work and reduced time at work.

I’ve managed to do so much more than I would if I was in the classroom.

You wouldn’t normally have the kind of time to learn all about that, develop skills, that’s been quite good.

Definitely feel like I don’t have the same pressures.

These quotes above from teachers show an awareness and appreciation of the advantages of not being physically in class with students. Kim and Asbury’s (2020) study that focused upon teachers’ experiences, identified factors that mediated against pandemic working conditions including supportive professional relationships that extended to the wider community of teachers together with stronger relationships with families and young people. Teachers reported being less busy and pressurized with extra time for planning. Another study focused upon the experiences of senior education leaders who cited the benefits of flexible working as: improved staff morale and HWB; skills development, and team-working/sharing practice (Cooper Gibson Research, 2020). For these educational leaders, additional factors included recruitment of skilled staff, succession planning at leadership level and development of strategic capacity. One participant in our study identified advantages of remote learning during the pandemic:

…definitely the pros have outweighed the cons and where we are as a school now, taking those couple of weeks to just kind of really get down and kind of in about it, our school is, honestly, everybody is amazing [laughs] at it, and how much they’ve embraced it.… the amount of work that I’m, and kind of learning that I’m providing the children has lessened down to essentially half an hours’ worth of work a day for the children, compared to nine to half two normally, so that has been easy.

However, remote working was also challenging to some teachers. Greenhow et al. (2021) highlight a contradiction: while calling it ‘unsurprising’ that teachers in the United Kingdom needed more training in digital pedagogy due to the rapid shift to remote learning, ‘in response to the overnight switch to remote teaching, teachers rapidly developed skills and adapted pedagogies’ (p.13). Several studies show that remote teaching was often difficult and stressful during the closure of school due to the pandemic (McLennan et al., 2020; Brown et al., 2021; Ferguson et al., 2021), “leading a heavy burden on teachers, who sometimes lack the social-emotional competencies to cope with such circumstances” (van der Spoel et al., 2020, p. 624; see also Hadar et al., 2020). In Cooper Gibson’s study (2020) with senior education leaders, key challenges included: school/teacher capacity to carry out tasks, available digital resources and skills to use them, leadership and accountability, online meetings and methods of communication. Participants in our study expressed similar views but also recognized the challenges of competing demands at home, particularly for those teachers who are also parents:

I mean, I find that I’m working all the way through till like six o’clock. Okay I might take a break here and there, but it’s very busy because when the children are sending in comments or pieces of work, I think it’s really important that they know that I’m here.

It’s quite difficult because I sometimes feel like I’m ignoring everybody when I’m working. Like if the children want to play and I’m like, ‘I can’t, I’ve got to work’. I think that’s quite difficult. It’s easier being away and having home and work as separate completely.

In Scotland, some teachers volunteered to work in education hubs and in specialist provisions whilst others worked entirely from home. One participant had to take her children to work as she was working in one of education hubs.

My children have to come to work with me, cause if I’m on at the hub then they also have to come too.

#### Recognizing the Importance of Identity, Agency, and Boundary Setting

It is important to acknowledge how the sense of time and work/home boundaries have overlapped during the pandemic and the impact of these on the lived experiences of people depending on individual and work circumstances (British Psychological Society, 2020). Teachers with young children found home working difficult but for those who lived alone there were also feeling of isolation, loneliness and presenteeism (Beattie et al., 2021; Dempsey and Burke, 2021). Kim and Asbury suggest that concepts such as teacher identity, self-stories and sense of agency may serve as mediators against work/home demands during the pandemic (Kim and Asbury, 2020). In their study, teachers cited the need for planning, caring about pupils, interacting with others and doing their job (see also Zuo et al., 2020, who argue that one innovation during COVID-19 was coordinating multiple institutions to share resources to support learning). They argue that it is important to understand how teachers have drawn meaning from their experiences during lockdown and how this may impact on education recovery over the next few years. One participant in our study reported that:

I’ve started my level 1 in BSL while still being able to mark and respond and give feedback to the pupils work immediately. It’s amazing.

However, several respondents highlighted the limitations of teaching remotely from home in terms of monitoring children’s well-being and supporting families:

It’s not possible to do everything that we do before, so daily we’d have emotional check-ins. We knew these children inside out… but we’re not able to support the families in anywhere close to how we were before…

Even just knowing what’s kind of going on in the children’s lives beforehand, you could tell if a child was hungry, you could kind of gauge by their appearance whether you’re needing to kind of give them stuff from our food bank, but now we’ve not got any idea, of kind of what’s going on there.

It’s nearly impossible to replicate what we’re doing in school when you’re online.

This concern of teachers for children’s well-being is reflected in the national GIRFEC practice framework in Scotland but it also aligns with bell hooks’ definition of self-actualization that views the body, mind, emotions as being interrelated. Her argument is that education traditionally tends to focus on the mind and gives little or no space to the body and emotions. Whilst GIRFEC’s eight well-being indicators are commendable in underpinning children’s well-being, learning and development in Scotland, the pandemic highlighted to many teachers the limitations of online teaching/working from home in terms of monitoring and safeguarding children’s well-being (we return to this limitation in the theme on teacher identity). Interestingly, in a study conducted by Vilchez et al. (2021) in California during the COVID-19 pandemic, it is argued that creating personalized and creative strategies as well as adequate support helped the development of students’ well-being.

#### Recognizing Protective and Risk Factors for Teachers Working From Home

Kim and Asbury (2020) and Collie (2021) have used the Job Demands-Resources Model (Bakker and Demerouti, 2007) to consider the factors that buffered or mediated the effects of job demands and home working on teachers during the pandemic. For example, Collie (2021) undertook a series of studies to explore teachers’ work-related experiences during the pandemic. She applies Job Demands-Resources theory (Bakker and Demerouti, 2007) to consider the impact of leadership factors as predictors of teacher resilience or workplace buoyancy as a personal resource around challenges of home working. The two roles of leadership are autonomy-supportive and autonomy-thwarting. A key finding indicated that autonomy-supportive leadership mediated work challenges for staff and that workplace buoyancy increased as work demand reduced.

One participant in our study referred explicitly to the leadership style of her boss:

cause I kind of tried to make my day as a kind of normal school day and I wasn’t wanting to be contactable or wasn’t wanting to respond to things sort of at seven o’clock at night, whereas some of my colleagues were doing that, and actually it was the boss who said, he was like, ‘look, you cannae be doing that, you need to make a cut-off point.’

Using the same theoretical framework, Kim and Asbury (2020) identified six job demands that contribute to poor mental health and well-being (uncertainty, workload, societal perceptions of the teaching profession, concerns for others, health struggles and multiple roles with competing demands). Three resources that were found to promote positive health and well-being were social support, work autonomy and personal coping strategies. A sense of control and flexibility in working from home were viewed by some participants as protective factors. One participant in our study reported that:

I would say it’s had a positive effect; I’ve been able to do things, set everything up in the morning, put it all out before the children are even awake, get on with my own little tasks I’ve been able to do like Open University courses.

Better work/life balance…working fully shorter hours.

We started this section using the idea of ‘recognition’ from hooks and this theme focuses on moments of recognition as suggested by the data. Teachers shared with us their awareness and recognition of the lived experiences of the moment in relation to teaching and learning and their interviews highlight the complexity and the uniqueness of each teacher’s lived experiences of teaching at home and in hubs during the pandemic. The interviews indicate that teachers’ moments of recognition were also action based, both in their own development and more evidently when supporting students and their families, as has been discussed in the previous theme and will be elaborated further in the next Theme.

We were encouraged by many instances in the teachers’ interviews where they describe their actions, while working from home, which focused on finding a solution that provided ‘hope’ for their learners. bell hooks would say that these moments of recognition for some of our participants engendered a sense of agency, liberation and resistance, recognizing that teaching as work and vocation could look different in a good way. For others, however, reduced agency to monitor children’s well-being was a key concern arising from teaching at home. For hooks’, recognition is fundamental to resolution of issues yet recognition on its own, that is naming the problem, without ‘a constructive focus on resolution, …[can] take away hope’ (hooks, 2003, p. xiv). We will return to this point in the conclusion of this paper.

### Theme 3: Teachers’ Identities

The challenges of teaching from home were not insubstantial and presented a challenge to how the participating teachers viewed themselves as a classroom practitioner. hooks’ work is a constant renegotiation between, on the one hand acknowledging who one is, asserting particular identities, while on the other hand constructing and reimaging one’s own life and identities. This section tries to capture this struggle. As hooks (1984) writes, ‘the ability to see and describe one’s own reality is a significant step in the long process of self-recovery; but it is only a beginning’ (p. 24). For hooks, there is a difference between recognizing oppression and resisting it. Hooks constantly questions:

…how do we create an oppositional worldview, a consciousness, an identity, a standpoint that exists not only as that struggle which also opposes dehumanization but as that movement which enables creative, expansive self-actualization? (hooks, 1990 p. 15)

Yet hooks reminds and challenges us to see that identities should not be seen as entirely as personal attributes (see the title of the next sub-theme), reduced to solely the experience that people have of their race, class, and gender (and others). If this is the case, then we fail to acknowledge ‘the objective structures of inequality produced by specific historical forces (such as capitalist production relations) that mediate the subjective understandings of both individuals and groups’ (Jaramillo and McLaren, 2009, p. 29). This is evident in this theme where we see the tensions play out between claiming a particular identity as a personal attribute and how this can potentially become problematic if we do not consider the interactions of that identity with the structures that surround it.

This theme of classroom practitioner incorporated a sub theme of blurring of boundaries and uncertainty.

#### I Am a Classroom Practitioner

For some of our participants, the uncertainty of the situation they found themselves in led to a repeated statement of their role throughout the interview. This repetition of their view of themselves as a classroom teacher was striking in its strength, almost meant to remind or convince themselves of who they were and what their role was during this uncertain time.

My strengths are as a classroom teacher

I feel my strengths are as a classroom practitioner

And that’s where I, that’s where I prefer to be, is actually working in the classroom

I’ve got a quite, a quite a kind of distinct role within the school. Kind of, I don’t know, the experienced class teacher.

This repeated confirmation of identity as a classroom practitioner was further strengthened by reference to the tasks of the role, and a desire to continue these tasks and ways of working as far as possible. One of our participants talked about how she approaches her work as a teacher: ‘I’m quite a kind of stickler for knowing what I’m doing’ and comparing this to her colleagues ‘I think we’re tending to do the similar sort of, well like we’re, we’re kind of setting tasks on a daily basis.’ She talked about being very structured and organized in how she set the work in the online environment and trying to keep the same structure as she would normally have in the classroom space. One teacher described how her planning had evolved into a routine that she felt comfortable with as a teacher

But then also how it’s evolved as well, I’ve kind of, we’re sort of now into a routine of Monday/Tuesday is a kind of normal day in the fact that I would set maybe a literacy task and a numeracy task, a health and wellbeing activity, and maybe something else. Then a Wednesday, I’m doing a grid, a grid for the day, and we’re just calling it Rainbow Wednesdays, and it’s just like lots of sort of interesting things for them to try.

And then a Thursday, we’re doing topic ideal stuff on a Thursday. And then a Friday, it’s more of a kind of, like we’ll do a quiz and, well I’ll set a quiz and I’ll get them to try and make quizzes up or I’ll give them a, you know, just sort of fun kind of things, just to kind of try. And also, I’m trying to get them to do a diary entry ‘cause we always did diaries on a Friday at school. And some of them have been doing it, and some of them haven’t, but you know.

As a teacher, the sense of being able to impose some order onto a novel teaching and learning environment strengthened how she saw her identity as a classroom practitioner and also the work that she does as part of this. She talked about not being able to focus or concentrate for the first couple of weeks of lockdown and how she couldn’t make sense of things. Being able to relate to the same kind of approach as she did in the classroom, chunking the day and the week, and imposing an order onto her work, appeared to help her to make sense of what her role was. As mentioned earlier, hooks challenges us not to reduce our identities to solely personal attribute. This does not allow the acknowledgment of the structures and discourses that contribute to the construction of our identity but also to the inherent inequality that exists in the interactions between ourselves and the objects/structures around us. Foucault (1979) also reminds us that a sense of self cannot develop independently of such structures. Our beliefs around who we are as teachers comes from societal beliefs around what teachers ‘should’ do, and how schools operate in our society. In this research, our teachers attempted to make sense of what it meant to be a teacher when they found themselves in such an unfamiliar and uncertain space by appealing to their understandings of a ‘collective’ notion of what it meant to be a teacher. Spicksley et al. (2021) found a similar leaning toward a collective rather than a personal identity when they conducted a discourse analysis exploring teacher’s experiences during lockdown.

#### Boundary Blurring/Uncertainty

This translation of the classroom order into the online environment was problematic though and led to confusion and uncertainty around identity as a classroom practitioner. One teacher commented how

you were trying to do exactly what you’d do in the classroom, but in the home instead, just trying to lift one model into another and they weren’t going together?’.

In many respects, this echos Jones and Kessler (2020) view who argue that the complexity of separating out personal and professional identity during lockdown when these identities are so enmeshed is not possible. This teacher recognizes and names the tension of trying to integrate her personal identity in the home and her professional, classroom identity. It is almost impossible to do this when the two are so interrelated during the lockdown period. Although the literature in teacher identity in the lockdown is currently limited, other researchers have also found this theme (Kim and Asbury, 2020).

Later on, this teacher reflected on the difficulties she experienced when she tried to merge the classroom structure into the online environment

So, you know, I think for me, I needed for me to have that structure in my life, cause I’m quite, I need to, I need to have that structure, and I think I did do a sort of transferral, I was like, right, okay, well this is what my day on a Monday would look like at school, so therefore, this is what we’re going to do

And then:

I suppose that was a bit of an eye opener for me because it wasn’t any, I wasn’t giving them any more than I would expect them to do at school, but then obviously they’re not at school.

This teacher reflects on the challenges of being the same kind of teacher as she was in the classroom, also making reference to how she will need to be a different kind of teacher in the classroom when she returns. This presents a challenge to her identity as a classroom practitioner though which is yet to be resolved. On the one hand, she talks about teaching ‘in the same manner as I did before’ but then almost immediately presents a dilemma recognizing that ‘we’re not gonna be able to treat them as, you know, as the same learners as we had before…’ and grappling with the uncertainty that this brings ‘I’ll need to get straight in my head, well, where does the, where is the connection made…’. She seems to resolve this boundary blurring, at least temporarily, by recognizing her work as a teacher as being related to the health and wellbeing of the children.

Not only was there tension evident in the blurring of boundaries between identity as a teacher in the classroom and what this meant when the ‘classroom’ was online, but also in the boundaries between professional and personal life. One teacher talked about the ‘stress inducing…and anxiety-provoking’ nature of trying to carry out her role as a teacher but from the home. There was evidence in this theme of the clashing of professional and personal boundaries

It’s quite difficult because I sometimes feel like I’m ignoring everybody when I’m working. Like if the children want to play and I’m like, ‘I can’t, I’ve got to work’. I think that’s quite difficult. It’s easier being away and having home and work as separate completely.

This potential conflict between professional and personal boundaries resulted in some reflection on how the physical space interacts with identity. Some of this was expressed as frustration at how this was working out for both themselves as teachers but also for their children who were being expected to complete schoolwork from home in their bedrooms:

And although I’m a teacher, it doesn’t make me any better at being a teacher at home, because at home I’m a mum so, you know, I don’t change into teacher role, because that would be wrong as well and it just wouldn’t fit.

And, you know, your bedroom is the place that you play, it’s not the place you sit down to do schoolwork.

The boundary blurring also extended to different and tasks that teachers found themselves undertaking:

I mean, we’re delivering food boxes, you know. I mean, so we’re being the, the food boxes come from the council, arrive at school, and then teachers and pupil support assistants volunteer to distribute food boxes to families. So, you know, families seeing us in a different role as well, and caring for our families.

Again, this points to the potential resolving of the boundary blurring around identity as a classroom practitioner by focusing on wellbeing.

### Theme 4: Parental Engagement

It has been well established in the research that parental engagement in children’s learning has a positive impact on learner engagement and achievement (Fan and Williams, 2010; Goodall and Vorhaus, 2011). There is debate in the literature as to the definition of parental involvement, the differences between involvement and engagement and what they look like in practice (Goodhall and Montgomery, 2014). A continuum of ownership and agency has been suggested by Goodhall and Montgomery (2014) as a means of clarifying the shift of emphasis from parent-school interactions (involvement) to parent-child interactions (engagement). They suggest that although some families are less involved with school interactions that does not necessarily imply that they are less involved with their child’s learning as barriers of confidence, cultural expectations, own educational experiences, health or work could prevent the desired level of school interactions. Parental support has never been so important as during COVID-19 when schools closed around the world and teaching and learning moved online with the agency for supporting children’s learning shifting predominantly to parents however the Scottish Government (2020a) made clear that ‘parents and carers were not expected to be teachers, nor to home educate in the formal sense’ (p. 6). Greenhow et al. (2021) analyzed key news media publications in the United Kingdom and United States, and showed how news media in the United Kingdom portrayed this contradiction. While parents are portrayed as teachers and ‘homeschoolers’ (p. 15) they are also receiving messages that they are not responsible for teaching their children.

Engaging with the works of bell hooks and simultaneously reflecting about data with regards to parental engagement, two concepts continually came to our mind, that of conscientization and the banking concept of education. These two concepts run through all her works, and here one can immediately see the influence of Paulo Freire’s work on hooks. From the data, as will be shown below, we believe that there were potentially moments of conscientization of parents on their children’s learning during lockdown. The move from school and classrooms to online learning often was a revelation for parents on what children where learning. As our data focused on primary age, online teaching and learning was very often mediated through parents. Thus, giving them a unique view to learning that often is enclosed within the classroom. Regarding the banking concept that hooks writes about, similar to Freire, she argues this is the basis of much of our learning. The reference here is that students are containers of knowledge, being filled to be used when they leave school. This concept has been challenged during the pandemic as will be argued below.

#### Supporting Learning Online

Teachers reported a wide variation in parental engagement with online learning with some families taking it on board enthusiastically whilst others struggled to engage at all. Lack of Digital Technology, lack of parental skill or confidence and trying to balance work and home-schooling responsibilities were acknowledged as possible causes for some of the disengagement. These paralleled some of the themes found by Garbe et al. (2020) when they explored parents’ experiences of children’s online learning during the pandemic and is supported by other research (Abuhammad, 2020; Beattie et al., 2021). Bubb and Jones (2020) found parental engagement in their children’s learning during lockdown increased and was partially motivated by the insight parents gained into their child’s learning journey. In the current study there was a sense from teachers that initial engagement was high and then waned as lockdown continued and pressures on parents mounted. This can be understood through Bronfenbrenner (1979) ecological systems theory wherein changes in one part of a system impacts on that in other areas of the system.

Communication with families, particularly those that were perceived as vulnerable families, perhaps where there were known child protection concerns, emerged as a key theme. There was particular concern for children of vulnerable families who were not engaging and worry about the impact of poverty, including food poverty, financial circumstances, safety and domestic abuse. Having processes in place for promoted staff making contact when children of such families had not been ‘seen’ online allayed staff fears slightly. Other research has found similar concerns (Kim and Asbury, 2020).

#### Widening of Teaching and Learning Concepts

Teachers in the study aimed to keep contact with families and reduce the burden on parents, many of whom were home-schooling and working from home simultaneously, by setting tasks that could be done as a family experience. This was in recognition of the impact of pressure on parents’ own health and well-being and an awareness of being reliant on parents to support the delivery of online learning, particularly for younger children (Beattie et al., 2021). One teacher stated that ‘I made it very clear to the parents that we are a team in this.’

Inadvertently this led to a widening understanding of teaching and learning as something beyond the confines of curriculum syllabus and textbooks which contribute to the banking style of learning, to the development of communication, life and transferable skills which could be learnt through day-to-day experiences such as conversations at the dinner table, gardening, playing board games and baking. The following quotes illustrates this:

As a school community we are trying to encourage families to come together and to use the time you know communicate, play games with each other, get outside to exercise, we are not wanting to be onerous.

Made me appreciate how much children can get from activities that a lot of parents don’t see as learning … we don’t need to be sitting down at a table to, you know, to do some writing in order to develop your literacy skills.

I’m just gonna do that lesson that I did on that the last time, I’ll do that one, and I don’t think that’s good enough, and I think this time has allowed me to see lots of different creative ways.’

And I think, in some ways I think that she’s actually, maybe progressed further being at home because I’ve taken all the other distractions away that are in the classroom. They’re not there.

Weaver and Swank (2021) found parents to value these daily learning experiences reporting them to be motivating and facilitating of creativity while Bubb and Jones (2020) found pupils themselves reporting most enjoyment with alternative creative tasks. From the chaos of the pandemic, opportunities to influence the future of Scottish education are emerging (Wrigley, 2020; Beattie et al., 2021). Zhao (2020) calls for rethinking education, questioning the ‘what, the how and the where’ as well as the ‘by whom and when’ (p. 31) of teaching and learning to create ‘the best education opportunities for all children, instead of improving schools’ (p. 30). Perceptions of the teachers in this study echo this thinking.

There were tensions inevitably; on the one hand teachers worried about parents not having the knowledge and skills of the craft of teaching to scaffold learning appropriately nor the understanding of formative learning and of learning from mistakes whilst on the other hand many acknowledged that there were many ways that learning could occur.

What I’m really trying to say is, I’m trying to explain something sometimes as a science that’s an art. I think it’s working because we’ve got a relationship working.

For some teachers there were also pressure between managing the challenges of teaching online and managing the expectations of some parents as to the format of teaching. Some promoted staff reported discrepancies between good teaching and parental confidence in the teaching with a trend of higher parental confidence with higher teacher visibility regardless of the teaching itself.

I think what these parents are seeing is a lovely person on screen saying, ‘Hey everybody, good morning. I’ve had this for my breakfast this morning. I hope you have a brilliant day and this is a really good learning task. See you later.’ Right? Because then what happens is when he posts really rubbish worksheets with the wrong answers, people forgive him more because they have seen him that morning, whereas with my other teacher who isn’t even posting audio messages yet, the parents aren’t reassured because they’re not hearing her and they’re not seeing her.

The concern about parents needing to be upskilled extended to ability to engage with the IT being used for teaching and learning (MS Teams and Seesaw in the main). Again, this reflected many teachers’ self-concerns particularly if they had not previously been using such technology in their teaching.

#### Parent and School Relationships

Families and teachers were reported by many to be having similar experiences, feeling over- whelmed, anxious, uncertain and uncomfortable with the changes to their daily lives and expectations around learning. This commonality led to increased empathy with parents, a lowering of professional barriers and for many improved parental relationships as a sense of comradeship developed. Many teachers recognized the strength in this new type of relationship and spoke of wanting this to be nurtured further post pandemic. This necessitates ‘a change of mindset on the part of many staff, a move from seeing “teaching” as the sole preserve of school staff’ (Goodhall and Montgomery, 2014, p. 407) and the mindset of parents it can be argued, to that of a collaborative venture with shared agency in children’s learning.

The pandemic context has enabled a dropping of professional boundaries which are sometimes barriers to authentic home–school relationships (Bryk and Schneider, 2002). A refocus on everyone supporting each other as human beings first and foremost, to navigate the restrictions on daily life that all were experiencing to varying levels of detriment took precedence.

We’re kind of shepherding each other, cause we’re a whole flock together and that’s how we need to get through this.

Valuing relationships with parents through the pandemic has been common (Kim and Asbury, 2020) and the shift in agency of learning to be informed by home and school information rather than just school-based information has increased trust between parents and school staff (Goodhall and Montgomery, 2014). We note a move toward hooks’ conscientization as the voices of all parties is being recognized and valued more, possibly because of the loss of the boundaries that structures create.

Goodall and Vorhaus (2011) in their review of parental engagement stated ‘Teachers often lack the confidence and knowledge to work with parents, and schools do not always recognize or value the ways in which parents are already engaged with children’s learning’ (p. 6). Responses in our research illustrate that the pandemic has offered a catalyst for re-evaluating parental involvement.

A heightened sense of school community was a common theme with staff and parents stepping out of their usual ‘roles’ to improve the lived experiences of all, e.g., parents and staff delivering food packages. A recognition of the benefits of increased harmony between school staff and parents (Goodhall and Montgomery, 2014) and the need for this to be nurtured and continued post pandemic emerged and will be interesting to follow up on the reality once the usual structures and ways of being filter back into our lives.

#### Priority of Health and Well-Being

The importance of health and well-being emerged as a strong theme with teachers recognizing the role that families had in promoting this. Teachers perceived the stress and anxiety of the pandemic context as being a major barrier to learning and recognized the need to address this first and foremost. Learning through daily interactions and everyday tasks within family life was deemed to be a more valuable way of keeping learning live whilst supporting family connectedness. This ties in with the widening concept of teaching and learning. There was recognition that family connectedness could be a protective factor for children and young people’s health and well-being and teachers saw ‘family’ tasks as being a means of providing this.

Wellbeing is the most important. Yes, children are missing out on learning but I think the learning is taking a different shape and I think it is more that the relationships with families that we have to think about and for what the children really capable of.

Other Scottish research on teachers’ lived experiences of the pandemic concurs (Beattie et al., 2021). In the Weaver and Swank (2021) study parents reported higher quality family interactions during lockdown and a desire for these to continue.

This theme highlighted instances of increased conscientization particularly on behalf of teachers about parents and the challenges faced at home. The compartmentalization of their input with children was removed when schools closed and the ensuing vagueness enabled an awareness and a listening to the other which was not possible before. As shown above, this changed the kind of teaching and learning that was expected, so that from the banking style of learning, teachers encouraged learning which was more about being together and about the attitudes which were thought to support children and families in this unprecedented time. The heightened awareness of the situations of parents and children enabled a new kind of listening to their voices and an appreciation of their participation in this new learning process.

### Limitations of the Study

We gathered contextual data from participants by asking them ‘tell us a bit about yourself.’ This yielded information about designation (Class teacher, Head Teacher, etc.) of the participants and the number of years of teaching practice, etc. While a key theme was teacher identity, we did not set out to gather specific sociological demographics of the children and families that teachers work with. In turn, we did not draw conclusions about how certain contexts within which teachers work might influence their identity.

As the invitations to participate in this study was done through different social media platforms and the interviews were carried out through MS Teams, we are aware that this implies teachers and Headteachers had to have access to technology and access the different platforms. While this is a limitation, given that the research started from the second week of the first lockdown, social media platforms and video conferencing were the only way we could communicate with teachers and Head Teachers as schools and Local Authorities were closed and no face-to-face encounters was possible.

Our study was a snapshot in time during the pandemic and therefore exceptional and atypical in teachers’ working experiences (Wong, 2020) thus making it unique to these circumstances. While the study is based on self-report during this time and is therefore is an appropriate research strategy (Collie, 2021), additional research may want to follow up participants’ journeys and changing identities and experiences as the lockdown continued to provide an alternative picture of the Scottish teachers’ lived experiences akin to van der Spoel et al. (2020) research with teachers in the Netherlands at two time points. Our study offered an in-depth exploratory study of primary teachers’ experiences during the pandemic, but future research may need to focus upon larger-scale and longitudinal studies that also include teachers from Early Years and secondary sectors (Greenhow et al., 2021). Similarly, this study did not look at how primary teachers from different year groups were living this experience. Focusing on teachers according to their year group would have given us different set of data. Future research might investigate this and retrospectively explore the lived experience of teachers.

Our paper considered emerging themes through the lens of bell hooks. Future research could make use of different theoretical frameworks such as activity theory to consider the ways in which different educational systems have interacted with each other during the pandemic. This approach would also be useful to consider systemic contradictions experienced by teachers in the changing context of work locations as they return to face-to-face teaching (Greenhow et al., 2021).

## Conclusion

We have used a theoretical framework inspired by bell hooks to explore and deepen our understandings of the experiences that teachers in Scotland reported to us in this research. It provides a unique exploration of a time and space in Scotland during 2020 that we were keen to capture. The taken for granted barriers which structure functioning within schools were removed and there seemed to be tentative exploration of possibilities, as we have shown above. Popa et al. (2020) refers to the opening of “Pandora’s box” (p. 8726) so that even after the moment of pandemic crisis is over, what has been unleashed will still need to be contended with, whether that is positive or challenging. Although the research is based in Scotland, the themes and understandings that emerged are of relevance to educators internationally. Schools across the world were impacted by various lockdowns imposed by the COVID-19 pandemic and teachers faced a common set of challenges that required to be navigated. “The ‘new normal’ has been announced and has already started in some contexts, but it also brings with it a number of challenges.” (Flores, 2020, p. 297).

Our research focused on four themes: change in pedagogy, agile and flexi working, teachers’ identities and parental engagement. In the first theme we write about the creativity required from teachers after school closure, and how this creativity was transformative in itself, causing teachers to question assumptions and systems and to feel that they were not going to be the same professionals even after school closure had ended. The second theme, about agile and flexi working, concerns the difficulties of feeling constantly ‘switched on’ and issues arising from the necessity of working from home. The following theme about teachers’ identities addresses the challenges to identities that our participants reported with the blurring of boundaries, making them uncertain, and how teachers engaged with such challenges and negotiated them. Parental engagement constituted the fourth and final theme, and talked about how learning from home needed support, about the impact of this on relationships, and about the necessity to revisit ideas of teaching and learning to incorporate the possible benefits of learning from home.

Within and between these themes, we found bell hooks’ ideas around engaged pedagogy to be a helpful framework to deepen the meanings that we were taking from our interviews. The idea of boundaries wove itself throughout our data as teachers expressed how the transgression of boundaries was occurring in multiple ways. We saw the exploration of the boundaries between pedagogical spaces (classroom vs. online) professional and personal boundaries and the boundaries of the systems in which education takes place. This has both challenged as well as opened up space for ‘extra creativity’ and new possibilities.

This research has offered a unique insight into the lived experiences of primary teachers working in Scotland with the interviews offering teachers spaces in which they could pause and reflect on the unexpected changes happening in their lives. Several teachers reported that the interview was in fact a chance for them to reflect on their actions in that unique moment. To our knowledge this is the only research that occurred from the second week of the COVID-19 school closure in Scotland. While the data is localized and specific, we think it contributes to a more general discourse of teachers and teaching and learning. The research contributes to thinking of how the absence of the school building gave teachers the possibility to think creatively about engaging with their students and their families. It is very rare that school closure occurs to that extent and for such a long period of time, and this research captures this. This also has helped question the relationship of teaching and learning outside of the classroom and school space and how teachers make sense of this. Furthermore, this research contributes to a unique discussion about the issue of the private/public life of the teacher, where working from home, often from their kitchen tables, with the available resources, was a practice that all teachers were engaged in. It has also highlighted the carry over between a teacher’s personal life and her professional life, as the teachers’ own experiences had an impact on their perspectives of the families whose children they taught.

We see in our data evidence of a shift in practice not just in the way teachers are ‘doing’ education but also, perhaps, in the way that teachers are ‘being’ as educators as they adapt to different ways of knowing. It is a way of understanding teaching that allows a more engaged pedagogy to develop.

## Data Availability Statement

The original contributions presented in the study are included in the article/supplementary material, further inquiries can be directed to the corresponding author/s.

## Ethics Statement

The studies involving human participants were reviewed and approved by the University of Dundee, School of Education and Social Work. The patients/participants provided their written informed consent to participate in this study.

## Author Contributions

All authors listed have made a substantial, direct, and intellectual contribution to the work, and approved it for publication. Authors are listed alphabetically.

## Conflict of Interest

The authors declare that the research was conducted in the absence of any commercial or financial relationships that could be construed as a potential conflict of interest.

## Publisher’s Note

All claims expressed in this article are solely those of the authors and do not necessarily represent those of their affiliated organizations, or those of the publisher, the editors and the reviewers. Any product that may be evaluated in this article, or claim that may be made by its manufacturer, is not guaranteed or endorsed by the publisher.

## Appendix Interview Questions

Interview Question: What are educators’ experiences of teaching from home/in hub during the COVID-19 lockdown in Scotland?

1. A little about yourself
2. What about your class?
3. What is your role as a teacher during this lockdown? Are you supporting home learning, or are you supporting children in a hub?
4. How has this experience affected your ideas of teaching, learning and curriculum?
5. How have you adapted to this new teaching situation?
6. What learning have you tried to focus on with your class? How far ahead can you plan or are there key areas on the horizon?
7. Can you provide an example of something that was challenging and something which was relatively easy.
8. What is the role of parents/carers in learning during this lockdown?
9. What are your thoughts about the impact of this on pupil transitions?
